# A Novel Evolutionary Strategy Revealed in the Phaeoviruses

**DOI:** 10.1371/journal.pone.0086040

**Published:** 2014-01-21

**Authors:** Kim Stevens, Karen Weynberg, Christopher Bellas, Sonja Brown, Colin Brownlee, Murray T. Brown, Declan C. Schroeder

**Affiliations:** 1 Cell and Molecular Department, Marine Biological Association, Plymouth, Devon, United Kingdom; 2 School of Marine Science and Engineering, University of Plymouth, Plymouth, Devon, United Kingdom; Sheffield University, United States of America

## Abstract

Phaeoviruses infect the brown algae, which are major contributors to primary production of coastal waters and estuaries. They exploit a Persistent evolutionary strategy akin to a *K*- selected life strategy via genome integration and are the only known representatives to do so within the giant algal viruses that are typified by *r*- selected Acute lytic viruses. In screening the genomes of five species within the filamentous brown algal lineage, here we show an unprecedented diversity of viral gene sequence variants especially amongst the smaller phaeoviral genomes. Moreover, one variant shares features from both the two major sub-groups within the phaeoviruses. These phaeoviruses have exploited the reduction of their giant dsDNA genomes and accompanying loss of DNA proofreading capability, typical of an Acute life strategist, but uniquely retain a Persistent life strategy.

## Introduction

All viruses broadly follow one of two life strategies, Acute or Persistent [1], [2], [3]. Moreover, the switch from Persistent to Acute in animal systems underpins emerging new viral epidemiology, notable examples being influenza, measles and HIV [2]. This transformation is often triggered by viruses jumping from one species to another. Viruses that follow an Acute life strategy have characteristic features that associate them with a disease phenotype; high reproduction and mutation rates, and greater dependency on host population densities for transmission. Many animal viral infections that are responsible for emerging epidemic diseases follow this Acute infection dynamic that originated from a Persistent viral life strategist [2]. Despite their likely prevalence, Persistent viral life strategies are not well described. Persistence is defined as a stable coexistence in an individual host, seldom causing disease, and transmission is often from parent to offspring [1]. Phaeoviruses infect the Ectocarpales brown algae, which are major contributors to primary production of coastal waters and estuaries [4], and separated from the kelps around 100 Ma [5]. Viral infections in protists contribute significantly to the sheer abundance of viruses in our oceans [6], and have been shown to play important roles in some of the major oceanic processes, such as plankton mortality [7], [8], nutrient cycling and carbon storage [9], [10]. Their ubiquitous nature means that viruses affect every aspect of life in the marine environment, and their importance in such fundamental areas as evolution [3], [11], the global food web and even climate change should not be underestimated [3], [9], [12].

**Figure 1:**
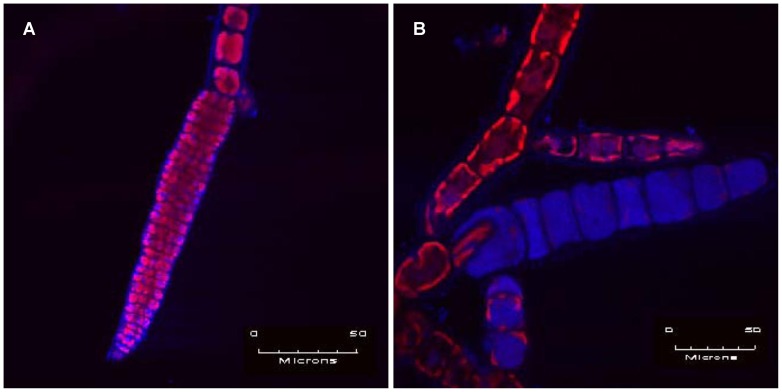
Epifluorescence microscope images of *E. siliculosus*. The pink stained individual spores (combination of DAPI stained blue DNA and red auto-fluorescence from nuclei and chloroplasts, respectively) are clearly visible within the normal zoidangium (A), whereas in (B) the zoidangium is misshapen and heavily stained showing that the space is filled with densely packed blue viral particles.

Protist viruses belonging to the family *Phycodnaviridae* [13] are members of the wider grouping of nuclear cytoplasmic large dsDNA viruses (NCLDVs). The coccolitho-[11], [14] and phaeoviruses [4], [15] are two examples of NCLDVs having opposing life strategies Acute vs Persistent, respectively. The former are lytic algal bloom terminators [14], while the latter covertly infect and integrate their genomes via the gamete and/or spore life stages of the host, forming a latent provirus which is transmitted to all cells during adult development [4]. As with most persistent viruses, phaeoviruses have no noticeable negative impact on the life-cycle of the host; however, overt symptoms of phaeovirus infection can be seen when the reproductive organs become deformed and produce virions, instead of gametes or spores (Figure 1).

To date, phaeovirus identity has only been confirmed for viruses infecting three species of filamentous brown algae: *Ectocarpus siliculosus* (Dillwyn) Lyngbye (Esil), *Feldmannia* sp. and *Feldmannia irregularis* (Kützing) Hamel (Firr); infected by EsV-1, FsV and FirrV-1, respectively [16]. They vary in genome size from 180–336 kb (Table 1). In addition, the genome of an *Ectocarpus* strain was found to contain a transcriptionally inactive copy of an EsV-1-like provirus [4]. Complete genome sequences show that EsV-1, FirrV-1 and FsV-158 contain a limited number of common single copy core genes, as well as many unique genes [15]. Five phaeoviruses, identified by morphology and life cycle, infecting *Ectocarpus fasciculatus* (Harvey) (Efas), *Feldmannia simplex* (Crouan & Crouan) Hamel (Flex), *Hincksia hincksiae* (Harvey) Silva (Hinc), *Pylaiella littoralis* (Linnaeus) Kjellman (Plit) and *Myriotrichia clavaeformis* (Harvey) (Mcla) have also been described in the literature (Table 1) [16]. Here we report on the phylogenetic placement of these phaeoviruses, using single and multi-gene phylogenies for three NCLDV core single copy genes, namely the major capsid protein (MCP), DNA polymerase (DNApol) genes, and a hitherto untested viral superfamily III helicase (VACV D5-like) gene.

**Table 1:** Ectocarpoid strains used for phaeovirus screening (adapted from Schroeder [16]).

Strain	Species	Family	Location	Genome	Number of sequence variants *			Concatenations**
				kb	DNApol	MCP	Helicase	
Esil	*Ectocarpus siliculosus*	Ectocarpaceae	New Zealand	336	1 (1)	1 (1)	1 (1)	1
Efas	*Ectocarpus fasciculatus*	Ectocarpaceae	France	320	2 (2)	1 (1)	2 (2)	2
Plit	*Pylaiella littoralis*	Acinetosporaceae	Alaska	280	1 (1)	1 (1)	1 (1)	1
Hinc	*Hincksia hincksiae*	Acinetosporaceae	France	240	1 (1)	-	2 (1)	-
Mcla	*Myriotrichia clavaeformis*	Chordariaceae	Argentina	320	1 (1)	2 (2)	-	2
Firr	*Feldmannia irregularis*	Acinetosporaceae	Canary Islands	180	2 (2)	3 (2)	2 (2)	4
Flex	*Feldmannia simplex*.	Acinetosporaceae	Ireland	220	9 (8)	6 (4)	8 (3)	22

: variant in DNA sequence (HG003317 - HG003355) with amino acid variation indicated in parentheses. A negative PCR result is indicated by a minus symbol.

: possible permutations for DNApol and MCP as seen in Figure 4.

## Results and Discussion

**Figure 2:**
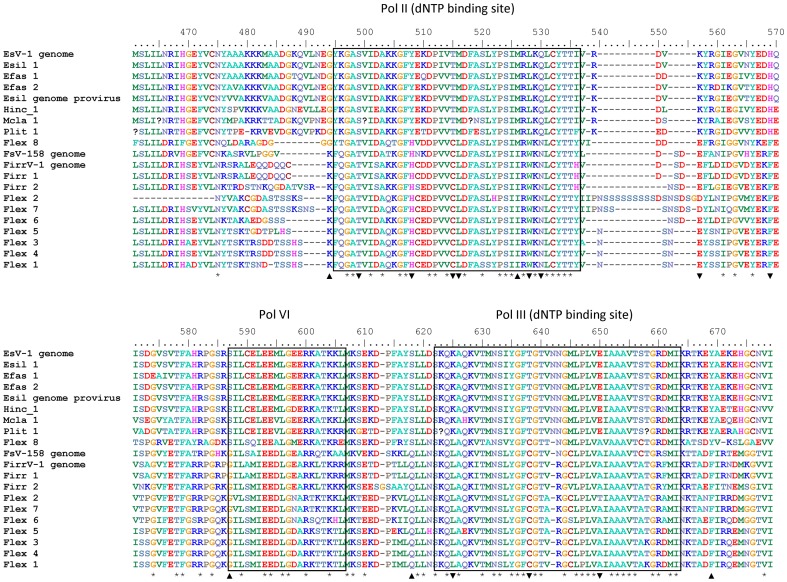
Partial nucleotide alignment of cloned fragment of the viral DNA polymerase gene. Numbers refer to amino acid position in the complete EsV-1 DNA polymerase gene taken from Delaroque *et al*. 2001 [20] (GenBank accession number NC_002687.1). Boxed regions indicate conserved polymerase domains [30]. * indicates conserved positions between all sequences, ▴ shows where the Flex 8 variant shares an amino acid with the larger viruses of sub-group A, ▾ shows where the Flex 8 variant shares an amino acid with the smaller genomed viruses of sub-group B.

**Figure 3:**
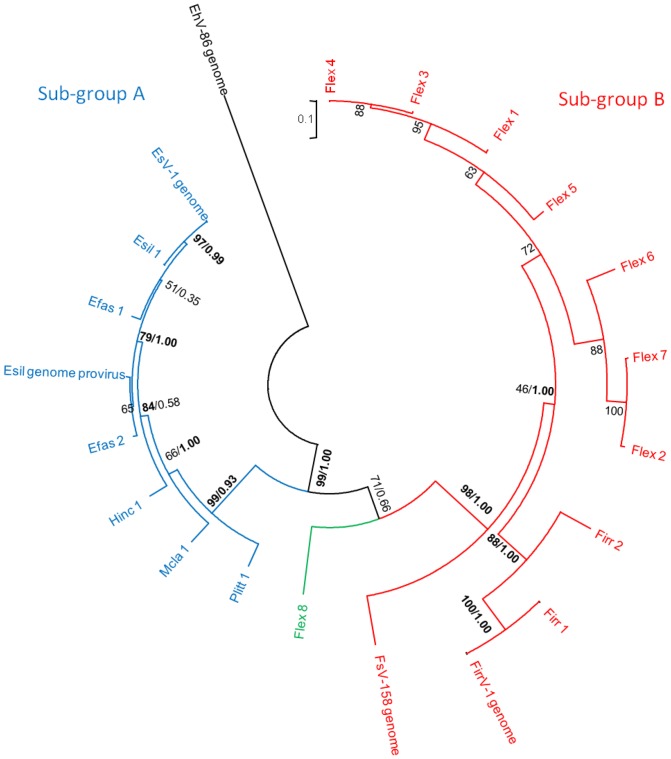
Maximum Likelihood analysis between variants of the phaeoviral sequences of DNA polymerase. Single value node labels represent ML bootstrap values. Where nodes are labelled with two values, this indicates that both ML and Bayesian topologies agree (whole numbers represent ML bootstrap values, decimals indicate Bayesian posterior probability). Sub-group A viruses are labelled in blue, sub-group B viruses are red and the intermediate Flex virus variant is green. Bold values are those greater than 75% bootstrap or probability.

**Figure 4:**
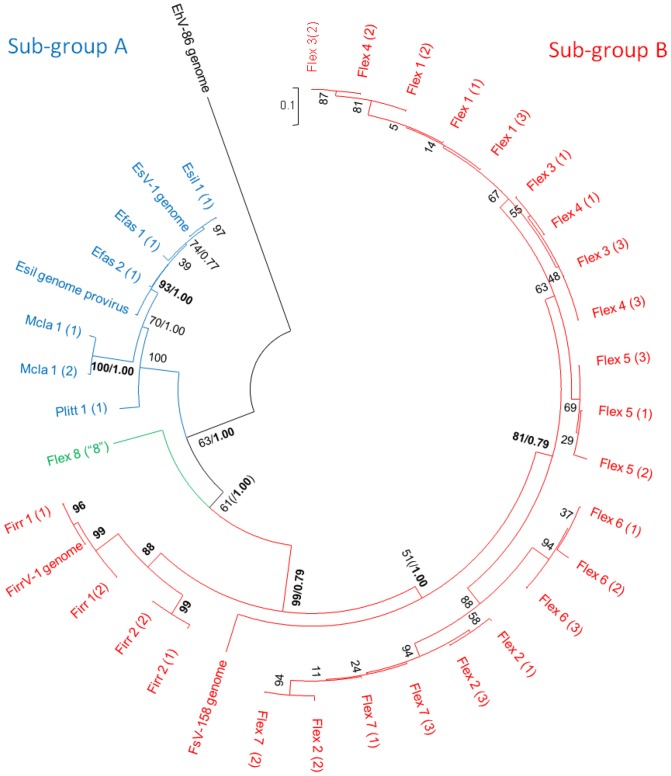
Maximum Likelihood analysis between variants of the phaeoviral sequences of concatenations of DNApol and MCP. Variants are labelled according to DNApol identifier initially, followed by the MCP variant number in brackets. In order to slightly reduce the number of combinations of sequences, where individual gene phylogenies show a clear separation of individual variants, these are concatenated together and excluded from the other combinations. Single value node labels represent ML bootstrap values. Where nodes are labelled with two values, this indicates that both ML and Bayesian topologies agree (whole numbers represent ML bootstrap values, decimals indicate Bayesian posterior probability). Sub-group A viruses are labelled in blue, sub-group B viruses are red and the intermediate Flex virus variant is green. Bold values are those greater than 75% bootstrap or probability.

EsV-1 is the only virus known to infect *E. siliculosus*, while Ivey *et al*.[17] reported the presence of two (and potentially four) different size variants (158 kb and 178 kb) of phaeoviruses in cultures of *Feldmannia* sp. Delaroque *et al*. [18] reported an incomplete FirrV-1 genome, with no evidence of multiple variants, within *F. irregularis*. Our viral sequences from Esil matched perfectly with reference gene sequences for EsV-1 (Table 1 & Figure 2). Notably, no additional sequence variation for EsV-1 could be found. The other available DNApol gene sequences for the reference genome, FirrV-1, were identified within the *Feldmannia irregularis* (Firr 1) isolate (Figure 2); however, at least one additional variant could also be identified (Table 1). This result is the likely explanation for the inability of Delaroque *et al*. to assemble the FirrV-1 genome [18]. All the other ectocarpoid strains contained two or more viral sequence variants, with the *Feldmannia simplex* (Flex) isolate containing at least eight different variants (Table 1). Our Bayesian and Maximum Likelihood inference trees (DNApol or multigene) were largely in agreement that the phaeovirus sequence variants group should be split into two distinct sub-groups: a virus sub-group A that infect multiple species across three families of the Ectocarpales (Figures 3 & 4) and a second sub-group B containing members that infect the genus *Feldmannia*.

Furthermore, there are two unexpected observations from these phylogenies. Firstly, the Flex 8 variant shares features with both of the sub-groups, whilst, unsurprisingly, being more closely connected to sub-group B (Figure 3 & Figure 4). A closer look at the DNApol sequence (Figure 2) shows not only the overall conservation of amino acids (32%) across all the phaeoviruses and the wider eukaryote kingdom as a whole, but also how certain amino acids can be assigned to either sub-group A (triangles, Figure 2) or sub-group B (inverted triangles, Figure 2). Moreover, one important conserved region, Pol III dNTP binding site, is known to be important for genome stability [19]. Daee *et al.* found that a single amino acid mutation was associated with extreme rates of spontaneous mutation in yeast. Three amino acid polymorphisms in this region (Figure 2, positions 618, 621, 625) could be a contributing factor to the large number of variants observed amongst the *Feldmannia* viruses. The second key observation is that Flex 8, at the base of the sub-group B clades in all the phylogenetic trees, is probably the progenitor virus to the *Feldmannia* sub-group B viruses. This, therefore, gives us a unique insight into the emergence of a new phaeovirus sub-group, which is likely to be a result of the genome reduction of an ancestral member from sub-group A. Both FirrV-1 and FsV-158 [15] show the loss of the DNA proofreading exonuclease gene (EsV-126) known to be present in EsV-1 [20]. We therefore hypothesize that these genomic modifications could have resulted in the key life strategy shift, thereby utilizing the high mutation rates more associated with acute infections. When and how this happened is unclear; however, the expansion of localised genomic regions in poxviruses, causing gene duplications and mutations have been proposed to be a response to overcoming changing immune responses after a host switch [21]. Whilst gene duplications have not yet been discovered in the phaeoviruses sequenced thus far, these expansions are usually followed by a rapid gene reduction in order to minimise the burden of replicating and enlarged genome, therefore a similar mechanism may also be involved here.

**Figure 5:**
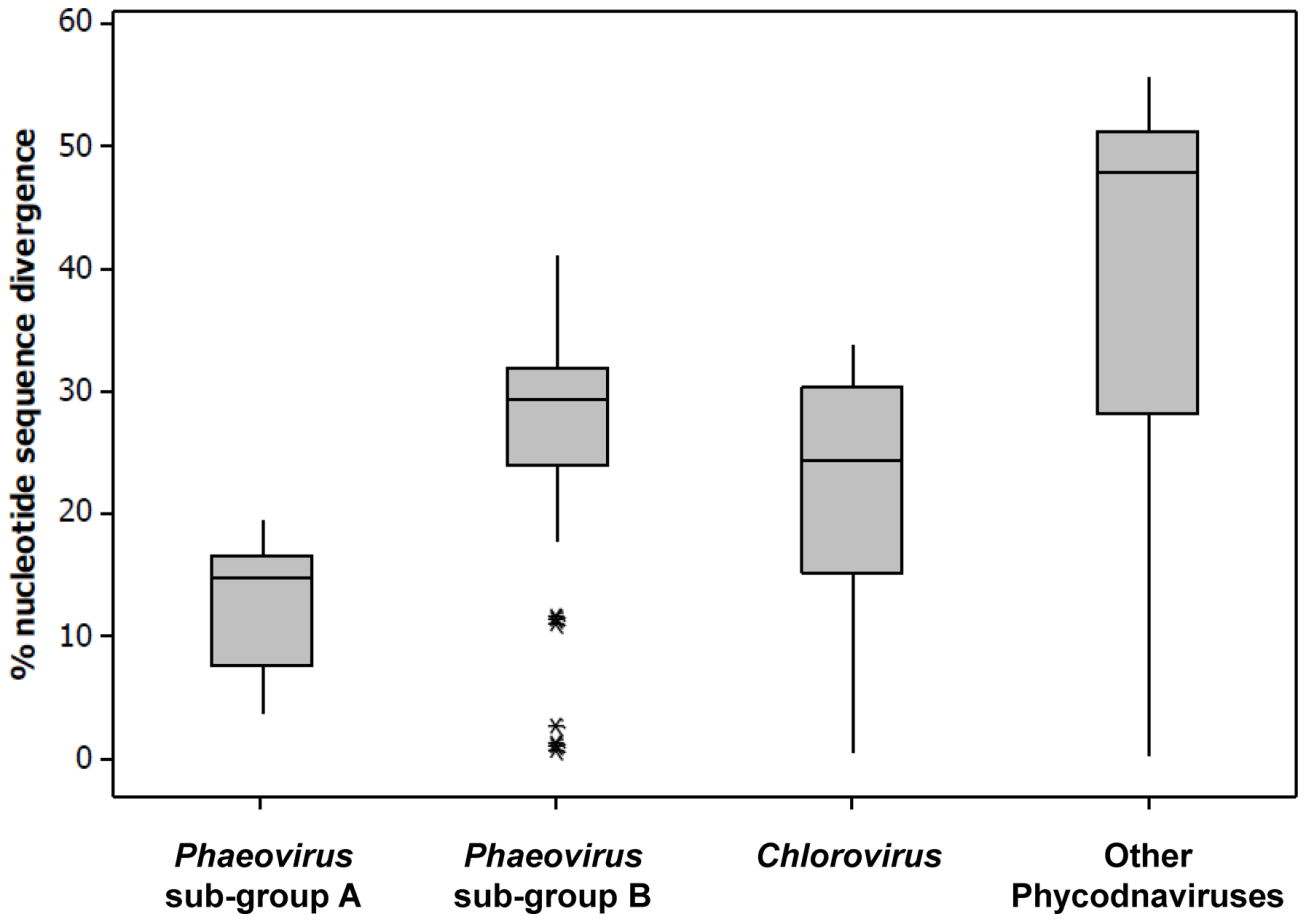
Box and whiskers plot of evolutionary divergence between nucleotide sequences of the DNApol. Identical sequences were not included more than once. The box represents the interquartile range which shows the middle 50% of the data, the bottom line being the first quartile, the middle line being the median and the upper line being the third quartile. The whiskers represent the maximum (or minimum) data point up to 1.5 times the box height above (or below) the top (or bottom) of the box. Outliers beyond the whiskers are shown as a *. Phaeovirus sub-groups are as shown in Fig. 3 & 4, with Flex 8 being included in sub-group B. Chloroviruses consist of thirteen viral isolates from *Paramecium bursaria Chlorella* (AF344202, AF344203, AF344211, AF344212, AF344215, AF344226, AF344230, AF344231, AF344235, AF344238, AF344239, M86837, U32985) and one from *Acanthocystis turfacea Chlorella* (AY971002). The other phycodnaviruses group consists of three viral isolates from *Emiliania huxleyi* (AF453961, AF453867, AF472534), three from *Micromonas pusilla* (U32975, U32982, U32976), five from *Ostreococcus tauri* (FJ67503, FJ884758, FJ884763, FJ884773, FJ884776), two from *Ostreococcus lucimarinus* (GQ412090, GQ412099), six from *Phaoecystis globosa* (A345136-AY345140, DQ401030), one from *Chrysochromulina brevifilum* (U32983), one from *Chrysochromulina ericina* (EU006632) and one from *Heterosigma akashiwo* (AB194136).

A pairwise analysis of the evolutionary divergence in nucleotide sequences within the various groups of phycodnaviruses (Figure 5) illustrates the shift by sub-group B to a genome characteristic of an *r*-like evolutionary strategy. Sub-group B has a median nucleotide divergence of 29.3% in the DNApol gene fragment, comparable to that of the other *r*- selected lytic phycodnavirus groups (24.3–47.9%). Sub-group A has maintained the classic *K –* selection life strategy with a much lower divergence of 14.9%.

There have been several studies that reported on the host specificity of phaeoviruses. EsV-1 can successfully infect *Kuckuckia kylinii* (Cardinal) producing virions infectious to the original host [22]. Other cross-species infections do not produce infectious virions although the virus does induce symptom-like deformities in the host, for example EsV-1 in *F. simple*x [22], or EfasV in *E. siliculosus* [23] and *M. clavaeformis* [24]. This demonstrates not only the potential of phaeoviruses to jump between species but also that not all jumps result in successful infections. Another example of this unsuccessful jump can be seen by the presence of an inactive provirus in the *Ectocarpus* genome [4]. Here we show that the provirus appears to be more closely related to an *E. fasciculatus* variant than to EsV-1 (e.g. Figures 3 & 4). This suggests that an *E. fasciculatus* virus infected an *Ectocarpus* species more closely related to *E. siliculosus* [25]. This study also confirmed the life-history and morphometric data that the viruses infecting Efas, Mcla, Plit and Hinc do indeed belong in the phaeovirus group. Moreover, there is also a corresponding grouping which can be created based on genome sizes (see Table 1); the larger viral genomes from Esil, Efas, Plit, Mcla and Hinc (240–336 kb) fall within sub-group A and the smaller viruses from Firr, Flex and *Feldmannia* sp [15] (158–220 kb) into sub-group B.

This study provides the first example of an emergent virus system retaining a Persistent life strategy, but exploiting an Acute strategist's high genomic mutation rate. Moreover, unlike current reports on how emerging acute diseases develop where cryptic persistent viruses cross species boundaries (e.g. HIV [26], H5N1 [27] and DWV [28]), which can have catastrophic consequences for new host survival, this study suggests a very different scenario of one in which the integration and diversification of Persistent viruses has been stably maintained over a long period of time. Similarly, due to their evolutionary link to animal viruses this infection strategy is likely to also occur in these systems, and further studies in this field may help our understanding of the spread of new emergent diseases.

## Materials and Methods

### Isolates & culture conditions

See Table 1 for a list of the phaeovirus-infected cultures used in this study. Each strain was cultured in a 40 ml petri dish at 15°C, 16:8 light-dark cycle, approximately 100 µmol photons m^−2^ s^−1^. The Western Channel Observatory (www.westernchannelobservatory.org.uk) is an oceanographic time-series and marine biodiversity reference site in the Western English Channel. In situ measurements are undertaken weekly at coastal station L4 (source of water for our study) and fortnightly at open shelf station E1 using the research vessels of the Plymouth Marine Laboratory and the Marine Biological Association. THE DATA POLICY of the NERC National Capability funded Western Channel Observatory is to make the data freely available at the point of delivery. Culture medium was filtered (30 kDa) natural sea water from the L4 sampling station close to the Eddystone Lighthouse near Plymouth, enriched with Provasoli's enrichment [29]. Sub-culturing into a new dish with fresh media was carried out every 14 days, when the cultures were pulled apart using forceps to separate out filaments in order to encourage production of zoidangia and virions.

### DNA extraction method

50–200 mg wet weight fresh algal material was transferred to an Eppendorf tube, frozen in liquid nitrogen and ground using Eppendorf grinders with 10 µl saturated ≤106 microns acid washed glass bead solution before proceeding with the Qiagen DNeasy protocol for Genomic DNA purification from cultured animal cells, starting with the proteinase K treatment. 40 µl proteinase K and 200 µl Buffer AL were added to the sample and incubated at 56°C for 30 minutes, before centrifuging for 2 minutes at maximum speed to separate out the beads. 200 µl ethanol was added to the resulting supernatant, vortexed and pipetted onto the spin column, to proceed with the first centrifugation step. For the final step, DNA was eluted using 100 µl water, instead of 200 µl in order to obtain a more concentrated sample.

### PCR, cloning & Sequencing

Degenerate primers were designed for three active viral genes (DNA polymerase (GRGGNCAGCAGATYAAGTG forward, GARTCCGTRTCSCCRTA reverse), helicase (GTGGCAGGTSATYCCYTTC forward, GTTKCCGGCCATGATYCC reverse) and major capsid protein (MCP) (CVGCGTACTGGGTGAACGC forward, AGTACTTGTTGAACCAGAACGG reverse)) against a consensus of published sequences from EsV-1, FirrV-1, FsV-158 and the provirus from the sequenced *Ectocarpus* genome. Degenerate PCR was carried out using Promega GoTaq® Flexi DNA polymerase kit, with an addition of 0.8 mg/ml bovine serum antigen (BSA). Cycling conditions were 95°C for 5 minutes, followed by 35 cycles of 95°C for 1 minute, a 30 second annealing step, an extension step at 72°C, and a final elongation step at 72°C for 10 minutes. Oligonucleotide and magnesium concentrations, annealing temperatures and extension times varied for each gene: DNApol required 1.25 mM MgCl^2^, 4 pmol/μl oligonucleotides, 50°C annealing temperature and 10 second extension time, MCP required 1.5 mM MgCl^2^, 8 pmol/μl oligonucleotides, 55°C annealing temperature and 30 second extension time and helicase required 1.5 mM MgCl^2^, 8 pmol/μl oligonucleotides, 55°C annealing temperature and 10 second extension time. Post-PCR samples were run on a 2% agarose gel at 80 V to achieve maximum separation between the bands. Samples with more than one product were purified by gel extraction; the band of the correct size was cut out of the gel and purified using the Qiaex II® Gel Extraction Kit. Samples with clean bands were purified using GenElute™ PCR Clean-Up Kit from Sigma. Purified PCR product was cloned into pCR®2.1, incubated overnight at 15°C before storing at −20°C until used. 4 µl ligation mixture was added to 0.2 ml competent cells and mixed. The cells were then incubated on ice for 40 minutes, heat shocked at 42°C for 2 minutes and returned to the ice for 5 minutes. 0.7 ml pre-warmed LB medium was added to the cells which were then incubated at 37°C for one hour. The cells were concentrated by spinning at 8000 g for 5 minutes, removing 0.5 ml supernatant, and re-suspended gently with a pipette before being plated out onto LB agar plates containing 5 µg/ml ampicillin, with 40 µl of 20 X-gal spread on each plate. Plates were incubated overnight at 37°C.

Single cloned colonies were picked from agar plates into individual 0.2 ml tubes containing 5 µl molecular grade water and heated to 95°C for 5 minutes to denature the cells before adding 10 µl 5× buffer, 5 µl 25 mM MgCl_2_, 5 µl 2.5 mM dNTPs, 2 µl each of 10 pmol/μl M13 forward and reverse primers, 0.2 µl Taq polymerase, 20.8 µl molecular grade H_2_O. Cycling conditions consisted of 30 cycles of 95°C for 45 seconds, 56°C for 45 seconds and 72°C for 45 seconds, followed by a final extension step of 72°C for 5 minutes.

PCR products were purified using the Qiaex II® Gel Extraction Kit and then sequenced using the BigDye® Terminator v3.1. The mix consisted of 3.5 µl 5× BigDye buffer, 1 µl Ready Reaction Mix, 2 µl template (6–14 ng μl^−1^ concentration), 1 µl primers (either M13 forward or reverse) at a concentration of 3.2 pmol μl^−1^ and 12.5 µl dH_2_O. Cycling conditions were 95°C for 2 minutes, followed by 30 cycles of 95°C for 30 seconds, 50°C for 30 seconds, 72°C for 30 seconds, then a final elongation at 72°C for 5 minutes. Sequenced reactions were precipitated by adding 5 µl 125 mM EDTA and 65 µl cold 100% ethanol and incubated in the dark at room temperature for 15 minutes. They were then spun for 30 minutes at 2200 g, the supernatant removed and the pellet washed with 60 µl cold 70% ethanol, and spun for a further 15 minutes at 2200 g. The supernatant was removed again and the pellet air dried. Sanger sequencing was carried out by Source Bioscience in Cambridge. Sequences were submitted to the European Nucleotide Archive with accession numbers (HG003317–HG003355).

### Phylogeny

Bayesian analysis of phylogenetic trees was carried out using MrBayes v3.2.1, running the analysis until the standard deviation of split frequencies reached <0.01 and the number of generations was >100 000. Maximum Likelihood analysis was carried out using MEGA5.1 WAG model with 500 bootstrap replications and the Nearest-Neighbour-Interchange heuristic method. DNApol and MCP sequences were combined in all possible combinations (Table 1) in order to create concatenations which were used to create Figure 4.

### Distance analysis

Nucleotide sequences were obtained for the various groups of phycodnaviruses that have been sequenced to data by carrying out a BLAST search of known genome sequences from each group. The phaeovirus sequences obtained in this study were split into two subgroups according to their phylogenies as shown in Figures 3 & 4. Chloroviruses were considered together with the prasinoviruses, and the remaining viral groups (coccolithoviruses, prymnesioviruses, raphidoviruses) were considered together since they are all lytic viruses of stramenopiles or coccolithophores. Pairwise distances were computed using Mega 5.05.
